# Developing dynamic defocus curve for evaluating dynamic vision accommodative function

**DOI:** 10.1186/s12886-022-02335-9

**Published:** 2022-03-05

**Authors:** Tingyi Wu, Yuexin Wang, Shanshan Wei, Yining Guo, Xuemin Li

**Affiliations:** 1 Department of Ophthalmology, Peking University Third Hospital, 49 North Garden Road, Haidian District, Beijing, 100191 P.R. China; 2 Beijing key laboratory of restoration of damaged ocular nerve, Peking University Third Hospital, Beijing, China

**Keywords:** Dynamic defocus curve test, Corrected dynamic vision accommodation, Dynamic visual acuity, Accommodation

## Abstract

**Background:**

To assess dynamic visual acuity (DVA) under different defocus statuses and explore the assessment of dynamic vision accommodation.

**Methods:**

Twenty subjects (6 males and 14 females) aged 18 to 35 were recruited. Nonmydriatic subjective refraction (sphere and cylinder) and accommodative tests including negative relative accommodation (NRA), positive relative accommodation (PRA), binocular cross cylinder (BCC) and accommodative facility using a flipper were performed. Binocular static visual acuity (SVA) and DVA at 40 degrees per second (dps) were measured under different defocus statuses (+1.5D to -4D in -0.5D steps) based on the refractive error fully corrected. Static and dynamic defocus curves were plotted. The area under the curve (AUC) and corrected dynamic vision accommodation (CDVAc) were calculated.

**Results:**

The study showed that the dynamic defocus curve fitted the cubic curve properly (*p*<0.001). DVA was significantly worse than SVA at all defocused statuses (*p*<0.001), and the difference was more significant at greater defocus diopters. Single factor analysis indicated that CDVAc was significantly correlated with NRA-PRA (*p*=0.012) and AUC_dynamic_ (*p*<0.001). Significant associations were observed between AUC_dynamic_ and PRA (*p*=0.013) as well as NRA-PRA (*p*=0.021). Meanwhile, DVA was positively correlated with PRA at 0D, -1.0D, -1.5D, -2.5D and -3.0D (*p*<0.05) and with NRA-PRA at 0D, -1.0D, -1.5D, -2.0D and -2.5D (*p*<0.05). Multiple factor regression analysis indicated that CDVAc (0D ~ -3.5D) and SVA (+1.5D ~ +1.0D & -2.5D ~ -4.0D) were significant influential factors for defocused DVA (*p*<0.05).

**Conclusions:**

Our study demonstrated that DVA had a defocus curve similar to that of SVA. CDVAc was feasible for the assessment of dynamic vision accommodative function. The dynamic defocus curve test could efficiently be applied in the evaluation of dynamic visual performance under different defocus statuses.

## Background

The accommodative function of human eyes enables us to clearly see the nearby visual targets; this function gradually decreases with age, leading to presbyopia [1, 2]. The accommodation tests currently used include accommodative amplitude, relative accommodation, accommodative facility (Flipper) and accommodative response [3]. Accommodative amplitude is the maximum power of focusing [4]. Relative accommodation tests evaluate the amount of accommodation to maintain a clear vision under a constant stimulus [5]. The accommodative facility is described as the ability to reverse the accommodation rapidly [6]. The accommodative response test yields the point at which the accommodative response is equal to the dioptric stimulus [3]. All of these tests mainly evaluate accommodative function by observing static optotypes. However, dynamic optotypes make up most real-life scenarios, and the dynamic vision accommodative function corresponding to dynamic visual acuity remains unknown.

Dynamic visual acuity (DVA) refers to the ability to identify the details of visual targets that have relative motion compared with the observers [7]. There is an increasing demand for DVA assessment, which is a relatively independent indicator in comparison with static visual acuity (SVA) and contrast sensitivity [8–10]. The dynamic visual acuity tests (DVATs) commonly used in clinical ophthalmology are based on a digital moving optotype demonstration with a computer screen at a fixed testing distance [11]. However, as the distances of observing moving targets in real life vary constantly, DVATs with a fixed distance may only yield limited information on dynamic visual function. Kinetic visual acuity tests can be used to assess the ability to identify visual targets approaching from a distant place [12, 13]. Due to the coupling of accommodation and convergence, an approaching optotype fails to accurately reveal the dynamic vision accommodation, and the readability changes as the optotypes approach during the test. Hence, an effective and convenient method to test the DVA under different accommodated statuses is currently lacking.

Static defocus curve tests (SDCTs) are traditionally used to evaluate the continuous visual performance at different distances [14]. Arranging various dioptric lenses on subjects’ eyes can induce a defocus status, which simulates different testing distances to evaluate the static visual quality [15], and phakic subjects can focus on the static optotypes through accommodation when adding positive or negative lenses to their eyes. SDCTs, which convert diopters to distances, have been shown to be a reliable method to demonstrate the static visual sensitivity at various accommodative statuses [16]. Thus, the DVA under different accommodative states can be measured similarly. Testing the DVA at different distances facilitates the exploration of defocused dynamic vision, which sheds light on the dynamic vision accommodation theory.

The present research combines SDCTs with DVATs to create a novel testing system, dynamic defocus curve tests (DDCTs). Moreover, we propose a new indicator, corrected dynamic vision accommodation (CDVAc), to assess accommodative function by observing moving optotypes based on the DDCT. The system intends to assess dynamic accommodative function and information on the DVA under various accommodative statuses that may assist a thorough evaluation of patients’ dynamic visual function and accommodation.

## Methods

### Participants

The present research was an experimental study on defocused dynamic vision and accommodation. The protocol was approved by the Ethics Committee of Peking University Third Hospital, and the research was performed in accordance with the Declaration of Helsinki. The entire research procedure was fully explained to all participants and written informed consent was obtained from each participant.

We enrolled subjects aged 18 to 35 whose monocular best corrected visual acuity (BCVA) was not worse than 0 (in logMAR). Exclusion criteria included those who had high myopia (>-6.00D), high astigmatism (>-2.00D), history of ophthalmic surgery, abnormal iris, vestibular dysfunction, and underlying ocular diseases such as strabismus, corneal diseases, retinopathy, neuro-ophthalmic diseases and glaucoma. Those who could not track dynamic optotypes, or obey the orders from the testers were also excluded from the research.

### Procedures

First, we collected general information, including age, sex, medical history and personal history, from the participants included in our study. Then, we conducted standard nonmydriatic subjective refraction to fully correct the ametropia with a phoropter (NIDEK CO., LTD., Japan).

Subsequently, the accommodation tests were performed in the order of NRA, the accommodative response test (binocular cross cylinder, BCC), PRA and the accommodative facility test. In the relative accommodation test, lenses were added in +0.25 steps gradually until the patients reported the first slight sustained blur. In the accommodative response test, additional positive or negative diopters were added until equal clear vertical and horizontal lines were noted by the patients. In the accommodative facility test, a ±2.00D flipper bar was applied with a testing distance of 40 cm. The patients were required to clear the 20/40 letters, and the bar was overturned to the opposite lenses once they could recognize the letters clearly. The number of cycles in 1 minute was recorded.

Next, in the SDCTs, a pair of additional lenses was added binocularly from +1.5D to -4.0D in 0.5D steps based on BCVA. Participants were required to identify the distant E optotypes of the phoropter at a distance of 5 m. The results were recorded in the form of logMAR.

### Dynamic defocus curve tests

The apparatuses used included a screen, a computer and a phoropter. We applied a twisted nematic screen with a DisplayPort 1.2 interface, refresh rate of 144 Hz, response time of 1 ms and luminance of 30 lux. The computer possessed a Thunderbolt™ 4 interface that could support the screen’s high refresh rate due to its high-speed data transmission. A custom-designed program was driven by MATLAB 2017b (MathWorks, Inc., United States) to generate dynamic optotypes according to the standard logarithmic visual acuity charts for optotype appearance and size arrangements. The visual angle of the moving optotypes presented at the testing distance was equal to the optotypes with the same decimal size on the standard logarithmic visual chart. The dynamic optotypes were designed to move horizontally from left to right in the middle of the screen. The phoropter was used to entirely correct refractive error before the test and to add additional diopters of spherical power to produce defocus during DDCTs. The detailed procedures of the DVAT can be found in our previous study [11]. Briefly, participants were required to sit at 3 m in front of the screen, and the velocity of dynamic optotypes was set at 40 degrees per second (dps). Binocular DVA was tested.

During pretraining, optotypes in size of 0.4 (in logMAR) were played randomly for five times. Participants were instructed to fully understand the movement pattern of the dynamic optotypes and were notified to distinguish the opening direction of the optotypes.

The formal test was continued based on the refractive error being fully corrected. A pair of +1.5D adding lenses were placed on participants’ eyes with the phoropter. The dynamic optotype with a random direction was played once each time, and participants were required to clearly state their judgment of the opening. Eight optotypes of a specific size were presented, and if the participants could correctly identify 5 out of 8 optotypes, then we changed the optotypes to one size smaller. The minimum size of the dynamic optotype (A in decimal) at which participants could recognize no fewer than 5 and the number (x) of optotypes recognized one size smaller than A were recorded. The final results for DVA were calculated as -log10A-b/80. After completing the DDCT with a +1.5D defocus status, we changed the lenses with a step size of 0.5D successively until -4.0D and repeated the procedures above.

### Corrected dynamic vision accommodation

We defined CDVAc based on DDCTs by reference to the essence of static vision accommodation (SVAc). It could be calculated as the dynamic vision accommodation (DVAc) corrected with undefocused dynamic visual acuity, as follows:

$$CDVAc=\frac{\mathrm{DVA}\mathrm{c}}{{\mathrm{DVA}}_{0\mathrm{D}}}$$

**Fig. 1:**
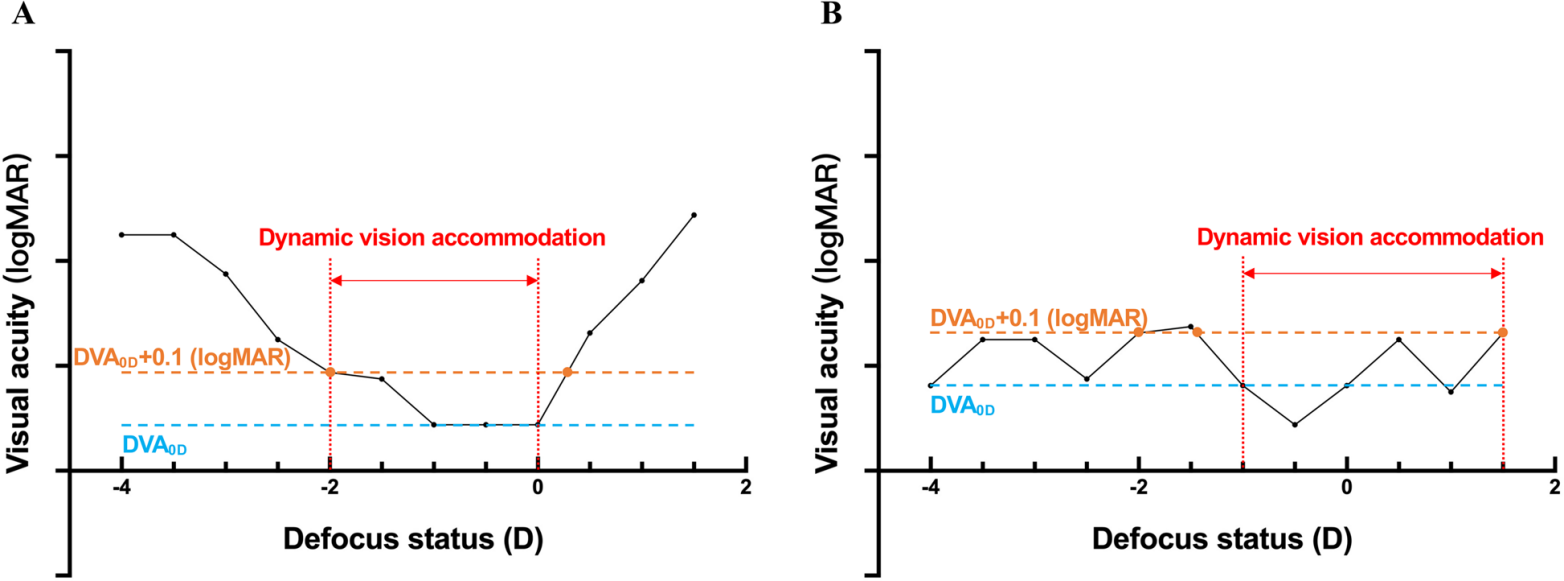
An example to measure corrected dynamic vision accommodation. The x-axis represents defocus status (diopter), and the y-axis represents visual acuity (in logMAR). The blue line represents the DVA of 0D, and the orange line represents DVA_0D_+0.1. The red line represents dynamic vision accommodation. The orange dots represent the intersections of the orange line and defocus curve. Note: DVA, dynamic visual acuity

DVAc was defined as the diopter range in which the subject’s DVA (in logMAR) was not worse than the DVA of 0D (without adding lenses) plus 0.1 similar to SVAc. Plus 0.1 was chosen based on our preliminary experimental results and calculation of SVAc. Defocused DVA was worse than DVA_0D_ in our pretest, and SVAc defined the diopter range in which the subject had the best SVA (plus 0). Thus, we chose the worse DVA closest to the DVA_0D_, which was plus 0.1. For example, as shown in Fig. 1A, the blue line represents DVA_0D_, and the orange line represents DVA_0D_ +0.1. There were two intersections (orange dots) between the orange line and defocus curve. The largest defocus point within the intersections was chosen as the upper limit of DVAc, and the smallest one was chosen as the lower limit. The range between these two limits was DVAc (the red lines). As shown in Fig. 1B, the two intersections closest to 0D were employed if there were more than two intersections.

### Statistical analysis

Statistical analysis was performed with IBM SPSS Statistics (version 26.0, IBM Corp., United States). The normal distribution of the data was checked by the Kolmogorov-Smirnov test. The results of continuous variables were recorded as the means ± standard deviation (*SD*). Static and dynamic defocus curves were drawn, and the area under the curve (AUC) was calculated with GraphPad Prism (version 9.0.0). The curve fitting of the dynamic defocus curve was accomplished by quadratic and cubic estimations.

One-way ANOVA test was used to analyze the differences in DVA under different defocus statuses. A paired t test was used to compare the DVA and SVA in all defocus statuses, as well as AUC_dynamic_ and AUC_static_.

The correlation between DVA and SVA in all defocus statuses, the DVA-SVA difference and defocus status, AUC_dynamic_ and AUC_static_, AUC_dynamic_ and static accommodation (including NRA, PRA, NRA-PRA, BCC and Flipper), DVA and static accommodation in all defocus statuses, AUC_dynamic_ and CDVAc, AUC_dynamic_ and DVAc, static accommodation (including NRA, PRA, NRA-PRA, BCC and Flipper) and CDVAc, as well as static accommodation and DVAc, were analyzed using Pearson or Spearman correlation analysis according to the distribution of the data (i.e., if there was a normal distribution, then Pearson correlation analysis was applied; otherwise, Spearman correlation analysis was employed)

A multivariate linear regression model was used to analyze the influential factors of AUC_dynamic_ and DVA in all defocus statuses. A collinearity analysis was implemented first. A variance inflation factor larger than 5 was considered to have multicollinearity, and factors were excluded from the model. The stepwise method was applied. The inclusion criterion was *F*≤0.05, and the exclusion criterion was *F*≥0.1. The level of statistical significance was determined as *p*<0.05.

## Results

### Baseline data

The demographic parameters are shown in Table 1. There were 6 males and 14 females included in this study. The average age of the participants was 26.15 ± 3.56 years.

**Table 1:** Baseline data

Demographic parameter	Average (mean ± ***SD***)	Range
**Age (years old)**	26.15 ± 3.56	20~34
**Average SE (diopter)**		
OD	-3.47 ± 1.91	-6.25~0
OS	-3.42 ± 2.03	-6.25~1.5
OU	-3.44 ± 1.91	-6.25~0.25
**Static accommodation**		
NRA (diopter)	2.08 ± 0.54	0.75~2.75
PRA (diopter)	-2.24 ± 0.78	-4.25~-0.75
BCC (diopter)	0.15 ± 0.25	-0.5~0.5
Flipper (CPM)	13.40 ± 3.05	7~19

Note: *OD* Oculus Dexter; *OS* Oculus Sinister; *OU* Oculus Unati; *NRA* negative relative accommodation; *PRA* positive relative accommodation; *BCC* binocular cross cylinder; *SE* spherical equivalent; *CPM* cycles per minute

### Static and dynamic defocus curve

**Fig. 2:**
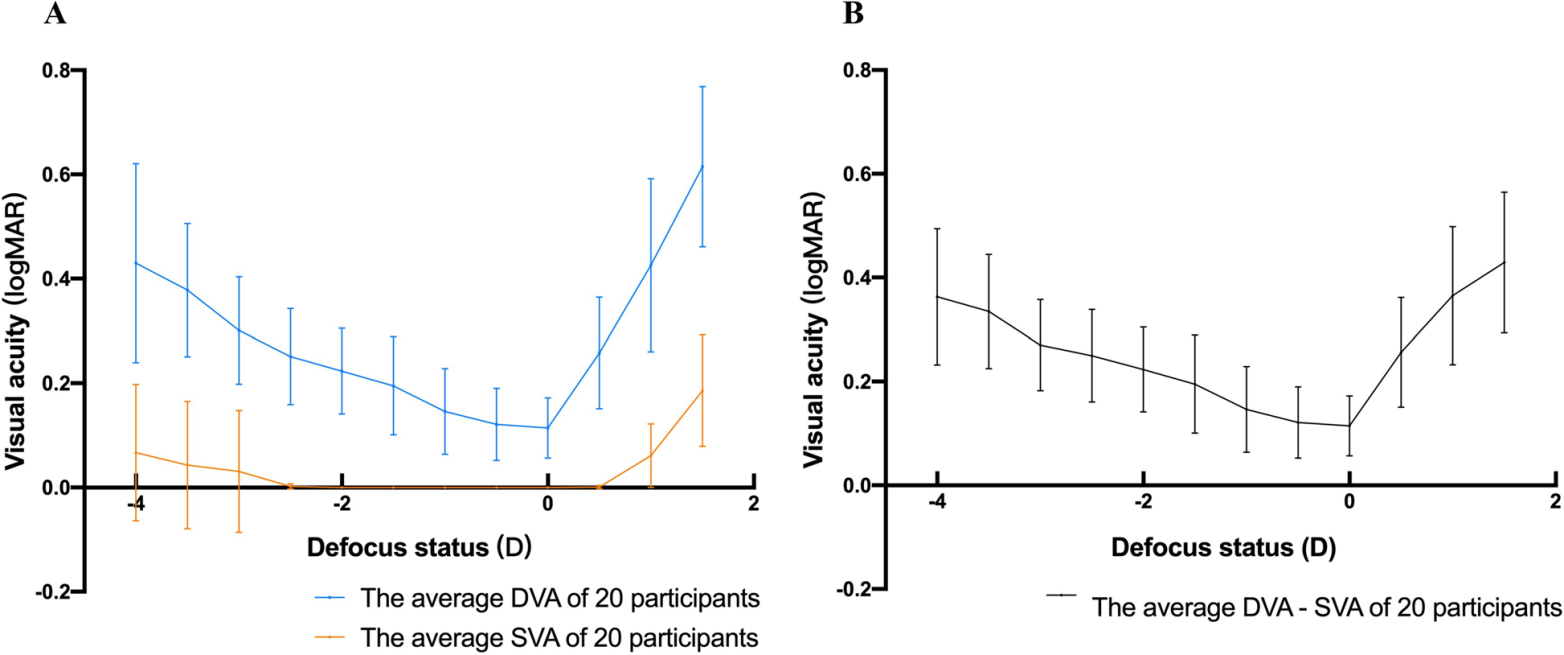
Binocular static and dynamic defocus curve. The x-axis represents the defocus status (diopters), and the y-axis represents visual acuity (in logMAR). **A** The blue curve represents the dynamic defocus curve (*n*=20), and the orange curve represents the static defocus curve (*n*=20). **B** The curve represents the DVA - SVA difference (*n*=20). Note: SVA, static visual acuity; DVA, dynamic visual acuity

The results of static and dynamic visual acuity with different adding lenses are summarized in Table 2, and static and dynamic defocus curves are shown in Fig. 2A.

**Table 2:** Binocular static and dynamic visual acuity

Defocus (diopters)	SVA (logMAR, mean ± ***SD***)	DVA (logMAR, mean ± ***SD***)	DVA - SVA difference (logMAR, mean ± ***SD***)
+1.5	0.186 ± 0.107	0.615 ± 0.154	0.429 ± 0.136
+1.0	0.061 ± 0.061	0.426 ± 0.166	0.365 ± 0.133
+0.5	0.001 ± 0.003	0.258 ± 0.107	0.256 ± 0.106
0	0 ± 0	0.114 ± 0.058	0.114 ± 0.058
-0.5	0 ± 0	0.121 ± 0.069	0.121 ± 0.069
-1.0	0 ± 0	0.146 ± 0.082	0.146 ± 0.082
-1.5	0 ± 0	0.195 ± 0.094	0.195 ± 0.094
-2.0	0 ± 0	0.223 ± 0.082	0.223 ± 0.082
-2.5	0.002 ± 0.005	0.251 ± 0.092	0.249 ± 0.089
-3.0	0.031 ± 0.117	0.301 ± 0.103	0.270 ± 0.088
-3.5	0.043 ± 0.122	0.378 ± 0.128	0.335 ± 0.110
-4.0	0.067 ± 0.131	0.430 ± 0.191	0.363 ± 0.131

Note: *SVA* static visual acuity; *DVA* dynamic visual acuity; *SD* standard deviation

The results showed significant differences in DVA under different defocus statuses (*p*<0.001). Curve fitting to the dynamic defocus curve showed that DVA varied cubically parabolically with increasing addition of adding negative lenses (quadratic, *R*^*2*^=0.85, *p*<0.001; cubic, *R*^*2*^=0.97, *p*<0.001).

The DVA at all defocus statuses was significantly worse than the SVA performance (*p*<0.001, see Table 2 and Fig. 2A) and showed a significant positive correlation with SVA (*r*=0.957, *p*<0.001). Differences between DVA and SVA in all defocus statuses were also calculated and are shown in Fig. 2B. The results indicated that the DVA-SVA differences were significantly correlated with the defocus status adding positive or negative lenses (*r*=0.987, *p*=0.013; *r*=-0.990, *p*<0.001, respectively).

The AUCs of the static and dynamic defocus curves in each patient were calculated. The average AUC_static_ and AUC_dynamic_ were 0.13 ± 0.15 and 1.47 ± 0.34, respectively. The results indicated that AUC_dynamic_ was significantly worse than AUC_static_ (*p*<0.001). AUC_dynamic_ was not significantly correlated with AUC_static_ (*p*=0.251).

### The influential factors of dynamic vision accommodation and the dynamic defocus curve

#### Single factor analysis of dynamic vision accommodation and the dynamic defocus curve

The DVAc and CDVAc were calculated with the formula above. The average DVAc and CDVAc were 2.18 ± 0.94 D and 23.64 ± 16.95 D. We analyzed the correlation between CDVAc and static accommodation, and the results (see Table 3) showed that CDVAc was significantly correlated with NRA-PRA but had no statistical correlation with NRA, PRA, BCC or Flipper. The relevance analysis indicated that DVAc had significant correlation with Flipper but had no correlation with NRA, PRA, NRA-PRA or BCC.

**Table 3:** Single influential factor analysis on dynamic vision accommodation and AUC_dynamic_

	CDVAc		DVAc		AUC_**dynamic**_	
	***r***	***p***	***r***	***p***	***r***	***p***
**Static accommodation**						
NRA	0.343	0.138	0.021	0.929	-0.118	0.619
PRA	-0.321	0.167	0.045	0.851	0.546	0.013*
NRA-PRA	0.552	0.012*	0.108	0.649	-0.511	0.021*
BCC	0.300	0.199	0.131	0.583	-0.086	0.718
Flipper	0.051	0.831	0.473	0.035*	-0.090	0.706
**CDVAc**					-0.797	<0.001*
**DVAc**					-0.424	0.063

Note: *CDVAc* corrected dynamic vision accommodation; *DVAc*, dynamic vision accommodation; *AUC* area under the curve; *NRA* negative relative accommodation; *PRA* positive relative accommodation; *BCC* binocular cross cylinder

The results of the single-factor analysis of AUC_dynamic_, which are listed in Table 3, showed that AUC_dynamic_ was significantly correlated with PRA, NRA-PRA and CDVAc but had no significant correlation with NRA, BCC, Flipper and DVAc. We also analyzed the correlation between DVA in all defocus statuses and static accommodative function, and the results (see Table 4) showed that DVA positively correlated with PRA at 0D, -1.0D, -1.5D, -2.5D and -3.0D, NRA-PRA at 0D, -1.0D, -1.5D, -2.0D and -2.5D, and NRA at +0.5D.

**Table 4:** Dynamic visual acuity and accommodative power

Defocus (diopter)	DVA & NRA-PRA		DVA & PRA		DVA & NRA		DVA & Flipper		DVA & BCC	
	***r***	***p***	***r***	***p***	***r***	***p***	***r***	***p***	***r***	***p***
+1.5	0.108	0.650	-0.072	0.763	-0.100	0.676	-0.083	0.727	-0.052	0.826
+1.0	-0.100	0.675	0.092	0.699	-0.032	0.892	-0.030	0.901	-0.169	0.476
+0.5	-0.290	0.215	0.100	0.676	-0.463	0.040^*^	-0.037	0.878	-0.321	0.168
0	-0.573	0.008^*^	0.547	0.013^*^	-0.304	0.193	0.327	0.159	-0.281	0.230
-0.5	-0.333	0.152	0.358	0.121	-0.031	0.898	-0.227	0.335	-0.345	0.136
-1.0	-0.506	0.023^*^	0.489	0.029^*^	-0.062	0.795	0.133	0.575	-0.073	0.760
-1.5	-0.583	0.007^*^	0.447	0.048^*^	-0.157	0.508	-0.094	0.695	-0.041	0.865
-2.0	-0.517	0.020^*^	0.438	0.053	-0.082	0.731	-0.167	0.481	-0.091	0.703
-2.5	-0.484	0.031^*^	0.516	0.020^*^	0.024	0.918	-0.117	0.622	-0.063	0.792
-3.0	-0.368	0.111	0.514	0.020^*^	-0.017	0.942	-0.166	0.485	0.012	0.959
-3.5	-0.257	0.273	0.442	0.051	0.213	0.367	-0.354	0.126	-0.045	0.852
-4.0	-0.183	0.441	0.394	0.085	0.269	0.251	-0.165	0.487	-0.074	0.755

Note: *DVA* dynamic visual acuity; *NRA* negative relative accommodation; *PRA* positive relative accommodation; *BCC* binocular cross cylinder

*indicates statistical significance

#### Multiple factors analysis on dynamic defocus curve

Collinearity analysis was implemented before a stepwise multiple linear regression analysis was performed, and AUC_static_ was excluded from the model from DVA_-3.0D_ to DVA_-4.0D_ as having collinearity with SVA (variance inflation factor>5). NRA, PRA, NRA-PRA, BCC, Flipper, DVAc, CDVAc, AUC_static_ and SVA were included in the model. The results (see Table 5) showed that AUC_dynamic_ was significantly correlated with CDVAc and AUC_static_. DVA significantly correlated with CDVAc from 0D to -3.5D and SVA at +1.5D, +1.0D, -2.5D, -3.0D, -3.5D and -4.0D.

**Table 5:** Stepwise multiple linear regression analysis of AUC_dynamic_ and DVA in all defocus statuses

Dependent variables	Independent variables	Unstandardized coefficient ***β***	95%***CI***	***p***	***r***^***2***^
**AUC**_**dynamic**_	CDVAc	-0.014	(-0.020, -0.008)	<0.001	0.661
	AUC_static_	0.852	(0.165, 1.540)	0.018	
**DVA**_**+1.5D**_	SVA_+1.5D_	0.731	(0.118, 1.344)	0.022	0.259
**DVA**_**+1.0D**_	SVA_+1.0D_	1.833	(0.825, 2.841)	0.001	0.448
**DVA**_**+0.5D**_	No independent variables were entered				
**DVA**_**0D**_	CDVAc	-0.002	(-0.003, -0.001)	0.002	0.531
	Flipper	0.009	(0.002, 0.015)	0.014	
**DVA**_**-0.5D**_	CDVAc	-0.003	(-0.004, -0.001)	0.004	0.382
**DVA**_**-1.0D**_	CDVAc	-0.003	(-0.005, -0.001)	0.007	0.341
**DVA**_**-1.5D**_	CDVAc	-0.003	(-0.006, -0.001)	0.004	0.374
**DVA**_**-2.0D**_	CDVAc	-0.003	(-0.005, -0.002)	0.001	0.481
**DVA**_**-2.5D**_	CDVAc	-0.004	(-0.005, -0.002)	<0.001	0.687
	SVA_-2.5D_	7.311	(1.758,12.865)	0.013	
**DVA**_**-3.0D**_	SVA_-3.0D_	0.537	(0.281, 0.793)	<0.001	0.687
	CDVAc	-0.003	(-0.005, -0.001)	0.003	
**DVA**_**-3.5D**_	SVA_-3.5D_	0.563	(0.199, 0.928)	0.005	0.556
	CDVAc	-0.003	(-0.006, -0.001)	0.018	
**DVA**_**-4.0D**_	SVA_-4.0D_	1.066	(0.569, 1.562)	<0.001	0.531

Note: *AUC* area under the curve; *DVA* dynamic visual acuity; *SVA* static visual acuity; *CDVAc* corrected dynamic vision accommodation

## Discussion

The DVAT is a promising test for evaluating the visual function in real life. The primary aim of this study was to further enrich the function of DVATs and explore dynamic accommodative vision function. Here, we introduced a novel method that can be applied to evaluate DVA under various distances and calculate CDVAc, thereby assessing dynamic vision accommodation.

In our study, we compared the dynamic defocus visual performances with its static counterpart. According to the theory of accommodation microfluctuation, when an observer focuses on a visual target, the accommodation of the eyes does not remain steady but fluctuates within a small range [17]. In a previous study, Iwasaki and Kurimoto [18] found that visual fatigue symptoms such as blurred vision were associated with this kind of accommodative oscillation. This might partly explain why DVA performances were significantly worse than SVA performances in the defocus status. The instability of accommodation may largely affect the ability to identify optotypes, and this effect might be augmented when the visual targets are moving. Furthermore, the greater the defocus that is present, the larger the differences between SVA and DVA. When the optotypes are static, participants may have time to accommodate an appropriate diopter for a clear vision. However, when the optotypes are in motion, it may be a possible that the optotypes pass through with an inappropriate accommodation, resulting in blurred vision. To conduct an overall evaluation of the DDCT, we drew lessons from SDCTs using the AUC for assessment [19] and observed that AUC_dynamic_ was significantly worse than AUC_static_, which was consistent with static and dynamic visual performances in the defocus status.

The power of accommodation can significantly impact the result of the dynamic defocus curve. In the present study, a correlation was found between AUC_dynamic_ and static accommodation (PRA and NRA-PRA). We also found that DVA was significantly or marginally correlated with PRA from 0D to -4D and NRA-PRA from 0D to -3D. As PRA and NRA-PRA refer to the adequacy of accommodation and NRA refers to the ability to relax the accommodation [5, 20], the results of our study indicated that the greater the amplitude of accommodation, the better the defocused dynamic vision would be. Previous research showed that benactyzine hydrochloride, an anti-cholinergic drug, can reduce the accommodative amplitude and DVA at the same time, which was similar to our results [21]. Regarding different defocus statuses, a negative defocus status causes images to focus behind the retina and then the observers need to accommodate to compensate for the defocus; in contrast, a positive defocus status induces images to focus in front of the retina, which is hard to accommodate by human eyes [22]. This may help to explain why no significant correlation was found between DVA and accommodation at the positive defocus status. A BCC was used to measure the accommodative stimulus at which the accommodative response was equal to the stimulus [3], and the blurry vision induced by the deviation of the accommodative response was identical at all defocus statuses. According to previous research by Locke and Somers [23], the accommodative response of young adults measured with the BCC test often ended with a small minus lens. This could be why AUC_dynamic_ and DVA had no correlation with the BCC in our study. Regarding accommodative facilities, the accommodation was substantially accomplished as soon as the defocus status was exerted. The identification ability relied on the maintenance of accommodation rather than the facility of accommodation.

CDVAc was a brand-new indicator to assess accommodative function, and its definition stemmed from the NRA and PRA. As relative accommodation meant the defocus range for maintaining the clearance of static optotypes [3], we designed CDVAc similarly attempting to quantify the ability to maintain the clearance while observing dynamic optotypes during accommodation. DVA is a multifactorial index, and disparity exists among individuals, although SVA was corrected to 0 (in logMAR). Thus, we normalized the amplitude of clear observations by the DVA at 0D to better reveal the accommodative ability. Consistent with our speculation, CDVAc rather than DVAc was significantly correlated with AUC_dynamic_. The results indicated that CDVAc was a promising index for predicting the accommodative ability of dynamic optotype observations. Additionally, CDVAc was also associated with NRA-PRA in the present research, rather than other static accommodative parameters. The amplitude of NRA-PRA depicted the ability to clearly view the static objects requiring accommodation. That is, the clearness of static optotype observations was an important factor for moving object identification in the defocus status. In multivariate analysis, CDVAc had a significant correlation with DVA at multiple defocus statuses, suggesting the superiority of its application in the evaluation of DVA under a defocus status over NRA, PRA and NRA-PRA. We also observed that SVA had a more significant relevance than CDVAc with DVA at a large defocus status. This is probably due to the DVA decline greater than 0.1 (in logMAR), which exceeded the range of CDVAc at the large defocus status.

Certain limitations are still present in our study. First, the sample size was too small, and only young subjects were included. Second, the mode of the dynamic optotypes used in our study was a horizontal movement that could not represent all of the moving visual targets met in daily life. Thus, more movement directions should be designed in future tests. Third, to fully cover the testing distances, the defocus status chosen in our study was +1.5D to -4.0D, which may take too much time to test the DVA. This may lead to visual fatigue and affect the accuracy of the results. Fourth, the velocity of the dynamic optotypes set in our study was 40 dps, which could not fully cover the speed frequently encountered in daily life. Fifth, we only considered accommodations in the present research. Vergence might affect accommodation as the observed distance changes, and further study is required to independently analyze the influence of vergence on DVAc.

DVA has a similar defocus curve to that of SVA. The introduction of DDCTs provides a possibility for a better evaluation of DVA at all defocus statuses. CDVAc defines the amplitude of maintaining a good DVA at the defocus status, which is a crucial indicator of the ability to observe the moving optotypes clearly with changing distances. DDCTs may be widely applicable in clinical ophthalmology and daily life. Nowadays, as cataract surgery mainly focuses on improving patients’ quality of life [24], the assessment of DVA after surgery is being given increasing attention. Traditional DVATs could only evaluate the distant visual acuity, while the novel DDCTs could evaluate all-distance dynamic vision, which could comprehensively assess the dynamic visual function of pseudophakic patients. Moreover, for patients implanted with functional intraocular lens (IOLs) including multifocal and extended depth of focus IOLs, DDCT might be more suitable for evaluating the continuous dynamic vision than the traditional DVATs. With further improvement, DDCTs can instruct cataract patients in the selection of IOLs based on dynamic vision at certain distances. Besides, DDCTs can potentially be applied to evaluations in certain specialty occupations that require observing moving optotypes, such as athletes and pilots.

## Conclusions

In summary, our study introduced a promising novel testing method to assess the DVA under various defocus statuses and a new indicator to evaluate dynamic vision accommodation. Specifically, DVA had a defocus curve similar to that of SVA. The DVA decreased as the defocus diopter increased, and the decline in DVA was larger than that in SVA. Our study also showed that SVA, PRA and NRA-PRA were the factors significantly affecting defocused DVA, AUC_dynamic_ and CDVAc. Multiple analyses showed that CDVAc had a significant negative correlation with defocused DVA and AUC_dynamic_, highlighting the potential for its application in dynamic vision accommodation evaluations. The DDCT resolves the unmet demand for assessing the DVA under different distances in clinical scenarios. Additionally, DDCTs and CDVAc lay an important foundation for further exploration of dynamic vision accommodation while observing moving objects. With further study, DDCTs and CDVAc are expected to undergo further improvements and might be applied in clinical ophthalmology for disease diagnosis and evaluation.

## Data Availability

The analysis data used in this study are available from the corresponding author upon request.

## Abbreviations

AUC: Area under the curve
BCC: Binocular cross cylinder
BCVA: Best corrected visual acuity
CDVAc: Corrected dynamic vision accommodation
CPM: Cycles per minute
DDCTs: Dynamic defocus curve tests
dps: Degrees per second
DVA: Dynamic visual acuity
DVAc: Dynamic vision accommodation
DVATs: Dynamic visual acuity tests
IOLs: Intraocular lens
NRA: Negative relative accommodation
OD: Oculus Dexter
OS: Oculus Sinister
OU: Oculus Unati
PRA: Positive relative accommodation
*SD*: Standard deviation
SDCTs: Static defocus curve tests
SE: Spherical equivalent
SVA: Static visual acuity
SVAc: Static vision accommodation
